# Investigating the role of RNA structures in transcriptional pausing using in vitro assays and in silico analyses

**DOI:** 10.1080/15476286.2022.2096794

**Published:** 2022-07-14

**Authors:** Simon Jeanneau, Pierre-Étienne Jacques, Daniel A. Lafontaine

**Affiliations:** a Department of Biology, Faculty of Science, Université de Sherbrooke, Sherbrooke, Quebec, Canada; b Centre de Recherche du CHUS, Université de Sherbrooke, Sherbrooke, Quebec, Canada

**Keywords:** Transcription, pause, hisL, RNA polymerase, Escherichia coli

## Abstract

Transcriptional pausing occurs across the bacterial genome but the importance of this mechanism is still poorly understood. Only few pauses were observed during the previous decades, leaving an important gap in understanding transcription mechanisms. Using the well-known *Escherichia coli hisL* and *trpL* pause sites as models, we describe here the relation of pause sites with upstream RNA structures suspected to stabilize pausing. We find that the transcription factor NusA influences the pause half-life at *leuL, pheL* and *thrL* pause sites. Using a mutagenesis approach, we observe that transcriptional pausing is affected in all tested pause sites, suggesting that the upstream RNA sequence is important for transcriptional pausing. Compensatory mutations assessing the presence of RNA hairpins did not yield clear conclusions, indicating that complex RNA structures or transcriptional features may be playing a role in pausing. Moreover, using a bioinformatic approach, we explored the relation between a DNA consensus sequence important for pausing and putative hairpins among thousands of pause sites in *E. coli*. We identified 2125 sites presenting hairpin-dependent transcriptional pausing without consensus sequence, suggesting that this mechanism is widespread across *E. coli*. This study paves the way to understand the role of RNA structures in transcriptional pausing.

## Introduction

RNA is involved in many crucial biological processes, such as amino acid transportation, ribosomal-subunit assembly, long non-coding RNA folding, riboswitch activity and intron splicing. All these functions are achievable thanks to the formation of RNA structures, which may be acquired cotranscriptionally [1]. To allow proper folding of nascent transcripts, the elongation of RNA polymerase (RNAP) is controlled by several cotranscriptional processes, such as transcriptional pausing [1]. A transcriptional pause consists of a temporary stop in the RNAP elongation process, giving substantial time for regulatory mechanisms to take place including ribosome binding site (RBS) sequestration [2,3], riboswitch folding [4] and transcription termination [5,6]. Transcriptional pausing is also important for the coupling of transcription and translation, limiting the presence of naked RNA between RNAP and the trailing ribosome, therefore controlling premature transcription termination [3,7]. Transcriptional pause sites are present on a large scale among prokaryotes [6,8] but they are also found in eukaryotes [9–11]. Transcriptional pause sites have been studied in vitro for decades using their half-life as a comparison metric [12]. In fact, the elongation complexes escape the pause site at a rate reflecting pseudo-first-order kinetics, or an exponential decay model, which allows convenient regression fitting and half-life computation [13,14].

Transcriptional pausing can be categorized into four distinct groups, such as elemental, backtrack, hairpin-stabilized and regulator-stabilized pauses [1,7,15,16]. The elemental pause mainly arises in a sequence-dependent manner and depends on interactions between RNA, DNA and the RNAP [15]. Following the formation of the elemental pause, the RNAP may translocate to upstream positions, thus giving rise to the backtrack pause. Alternatively, backtrack pauses can also happen when elongating RNAP reach an obstacle along the template that halts the progression of transcription. However, in the case of hairpin-stabilized pause, the formation of specific RNA structures, such as hairpins or pseudoknots, may allow the nascent RNA to interact with the RNAP and thus to prevent further RNAP elongation. Recent cryo-EM data showed that a portion of the RNAP partially rotates (swivelling movement) in the context of an hairpin-stabilized paused elongation complex, thereby creating steric clashes preventing transcription elongation [17]. Lastly, protein regulators, such as NusA and NusG, may also participate in transcriptional pausing by directly interacting with the nascent RNA and the elongation complex [15].

The roles of a handful of transcriptional pause sites were discovered in the last decades. Among these transcriptional pause sites, those by far the most studied are located in the *Escherichia coli* histidine and tryptophan operon leader peptides, namely *hisL* and *trpL* pause sites. Both pause sites were shown to rely heavily on cotranscriptionally folded RNA hairpins stabilization [18–21]. It has been shown that disrupting these RNA structures by point mutations prevents the pausing event in vitro [19]. Yet, transcriptional pausing is restored by rescuing the formation of the RNA structure through compensatory mutations. Moreover, both *hisL* and *trpL* pauses are enhanced by the NusA factor that is an essential transcription factor universally distributed among bacteria and archaea [22]. NusA is involved in both termination and antitermination processes [22–24] and has been observed to directly interact with the paused elongation complex and the upstream RNA hairpin, thus creating a bridge that forces the elongation complex to remain in a paused conformation [25].

Both *hisL* and *trpL* genes are located in leader regions of biosynthetic operons [6,26]. Their transcription is regulated in such a way that termination and antitermination signals are in constant competition depending on cellular availability of specific amino acids. To understand this regulatory mechanism, the RNA structure in both systems has been studied, uncovering a link between structure and pausing [19,20]. For both *hisL* and *trpL*, the link between RNA structure formation and transcriptional pausing was demonstrated using mutational analysis [20,26,27]. It was first shown in *trpL* that a unique RNA stem-loop structure is folding immediately upstream of the paused RNAP [20]. The duration of the *hisL* pause is strongly decreased when mutations are introduced within the stem, but not in the loop region [20,26–28]. Importantly, when compensatory mutations are introduced to rescue the formation of the predicted *hisL* RNA stem, the efficiency of the pause is restored to a level similar to the wild-type (WT) [25]. However, changing the size of the hairpin loop drastically decreases the impact of NusA on the pause half-life [25], suggesting that the loop shape, but not the sequence identity, is important for NusA-dependent pause stabilization. The importance of the hairpin has recently been highlighted by cryo-EM data showing that the hairpin is closely interacting with positively charged amino acids of the RNAP exit channel, therefore enhancing the duration of the pause [17].

Recent findings from high-throughput techniques such as *Nascent Elongating Transcript Sequencing* (NET-Seq) [29] suggest that the importance of transcriptional pausing has been underestimated. Indeed, the detection of several thousand pause sites using NET-Seq revealed that pause sites are not only restricted to translation start sites, but are rather widespread across the genome, suggesting that additional molecular mechanisms might be used depending on the genomic location [30–32]. The studies also revealed a DNA consensus sequence G_−10_Y_−1_G_+1_ where RNAP elongation is pausing before incorporating the G residue at position +1 [30–32]. It was found that the identity of G_+1_ is the most important element of the consensus for the pausing process [32]. It was also demonstrated that the sole presence of the complete consensus is sufficient for pause escape, suggesting that it represents a minimal system for transcriptional pausing [30]. Lastly, the relationship between potential upstream RNA structures and the DNA consensus has not been studied yet, leaving the context of these pauses completely unknown.

While the vast amount of knowledge about transcriptional pausing has been generated using in vitro systems relying on *hisL* and *trpL* pauses as models [18,25,33], there are additional transcriptional pauses that have been previously reported and that are still incompletely characterized. For example, although the pauses in *rfaQ* (also known as *waaQ*) [16,34], *rnpB* [35,36], *leuL* [37,38], *pheL* [38,39], *thrL1* [38,40], *thrL2* [38,40], *pheM* [30] and *pyrL* [41] have been reported, no detailed mechanism has been deduced nor proposed for most of them, even if a nascent RNA structure was often suggested. In this work, we have investigated the importance of upstream RNA structures for transcriptional pausing and the respective roles of the RNA structure and DNA consensus for pausing. To achieve this, we investigated the presence of pause-stabilizing secondary structures using NusA and by performing a mutagenesis approach. We find that some of these formerly discovered – but still uncharacterized – transcriptional pauses (namely *leuL, pheL* and *thrL1*) are stabilized by an RNA secondary structure, while also presenting the consensus sequence. Furthermore, we also observe that less characterized pauses (*pheM, pyrL*, and *thrL2*) are unlikely to rely on an RNA structure to achieve transcriptional pausing. Our study also confirms previous hypotheses that transcriptional pausing within *rfaQ* and *rnpB* does not appear to rely on the stabilization of an RNA structure. Lastly, we evaluate the importance of hairpin-stabilized pausing among three Net-Seq datasets. Our results suggest that a whole group of pauses could depend, solely or with a consensus sequence, on RNA hairpin stabilization. The results presented here aim to further expand the repertoire of known RNA structures involved in transcriptional pausing and to consolidate the existing knowledge about the RNA structure and the DNA sequence consensus involved in transcriptional pausing mechanisms.

## Results and discussion

### Study of the well-known hisL pause site

**Figure 1.**
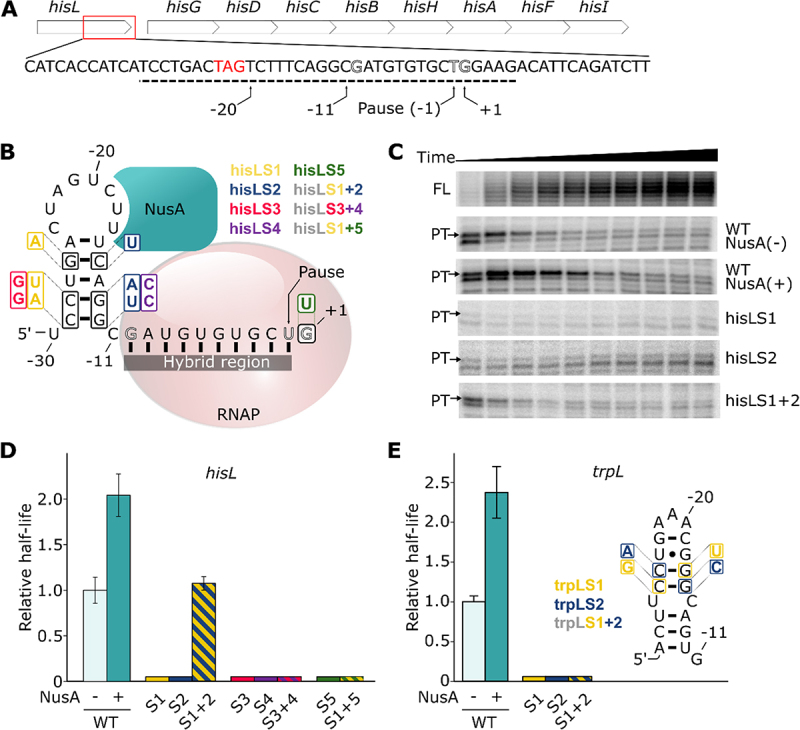
Transcriptional pausing at the *hisL* and *trpL* pause sites. (A) The *hisL* pause site is located in the *hisL* leader peptide of the *hisLGDCBHAFI* operon. The red rectangle represents the region of the sequence in which is found the pause site within the operon. The stop codon of the leader peptide is shown in red. The −1 position refers to the pause site. The dashed line represents the genomic segment used for in vitro transcription assays in this study. (B) Schematic of a cotranscriptionally folded RNA hairpin located upstream of the DNA-RNA hybrid region. The hairpin is shown interacting closely with exit channel of the RNA polymerase (RNAP, pale red). NusA (teal) can establish a bridge between the hairpin loop and the RNAP, resulting in an extended pause half-life. The consensus sequence G_−10_Y_−1_G_+1_ is presented in hollowed black residues within the hybrid region. The different mutant sets used in the study are shown and are colour coded. Double mutants (hisLS3, hisLS6 and hisLS8) are shown with colours corresponding to single mutants. (C) Representative transcription kinetics of the *hisL* pause. The full-length (FL, 77 nt) and the paused transcripts (PT, 54 nt) are shown. Only the paused transcripts are shown when using NusA and for the tested mutants. (D) Relative half-lives of *hisL* pause and the different mutants used. The mutants are depicted by their respective numbers (e.g. 1 for hisLS1). The colours are related to the panel B and hatched bars represent combinations of the corresponding mutation sets. (E) Relative half-lives of *trpL* pause and the different mutants used. The nomenclature used is the same as the one described in panel D. The predicted structure of the hairpin and the different mutants used are shown on the right. The compensatory mutant trpLS3 is shown with colours corresponding to single mutants trpLS1 and trpLS2.

The *his* operon leader pause was first observed in *Salmonella enterica* serovar Typhimurium [19]. Similarly to other biosynthetic operons regulated by attenuation such as *thr* [42] and *trp* [6,43], a strong pause is located in the leader region (Figure 1A). Transcriptional pausing at this position is proposed to facilitate the coupling between transcription and translation [7,44]. In fact, the pause allows sufficient time for an upstream ribosome to reach the RNAP and to regulate transcription elongation accordingly. In low histidine concentration conditions, the ribosome stalls at histidine codons in the leader peptide, resulting in a slower rate of translation elongation. In this context, the ribosome footprint prevents the nascent transcript to fold into a terminator stem-loop, hence allowing transcription of the downstream *hisG* operon. As a result, the *hisL* pause indirectly regulates the rate of premature termination and allows fine tuning of the operon expression. Importantly, the *hisL* pause contains the DNA consensus sequence identified to be important for transcriptional pausing [30]. These observations suggest a regulatory mechanism where both the nascent RNA structure and the DNA consensus sequence play a role in the pausing process.

To study the relative importance of the RNA hairpin and the DNA consensus for transcriptional pausing, we first performed single-round in vitro transcription assays using the *hisL* pause site as a model. The template consists of a promoter allowing the transcription of a short initiation and spacer sequences that are fused to the *hisL* pause sequence containing positions −30 to +5 relatively to the pause site (see schematic in Supplementary Figure S1). The initiation and spacer sequences were designed to fold into a stem-loop structure to prevent unexpected interactions with the downstream sequence. In this context, the nascent *hisL* RNA hairpin is expected to be located between positions −30 and −11 (Figure 1B). All obtained half-life values and associated characteristics are reported in Supplementary Table S1.

Synchronized transcription elongation assays revealed paused transcripts appearing at early time points and decreasing in intensity over time (Figure 1C and Supplementary Figure S2A). This migration product has been mapped to a 54 nt transcript, as expected from the pause site. The intensities of the pause and full-length transcripts respectively decreased after 30 s and increased over the course of the reaction (Supplementary Figure S2B). Consequently, the proportion of paused transcript decreased sharply in the first 100 s (Supplementary Figure S2C) and a non-linear regression analysis revealed a pause half-life of 8.5 ± 1.4 s for the *hisL* pause (Figure 1D). This half-life value is 5- to 10-fold lower than previously obtained values [25,45], suggesting an influence from the different experimental conditions used. For instance, these values were usually obtained with the use of a lower concentration of GTP (10 µM) during transcription, most probably resulting in a longer pause half-life since the position +1 corresponds to a guanine. Hence, the pause was determined to be less efficient at saturating GTP concentration, where its half-life is ~2 s [30].

We next performed in vitro transcription assays using the wild-type (WT) sequence in the presence of NusA. As expected, a half-life of 17.3 ± 2.0 s was observed, which corresponds to an increase of ~2-fold (Figure 1D) [46]. Together, our results confirm that the *hisL* pause is affected by the presence of NusA, consistent with a nascent RNA hairpin being involved in transcriptional pausing. Again, in other studies using lower GTP concentrations, NusA was shown to increase *hisL* pause half-life by ~3-fold [25] or ~4.4-fold [45], suggesting that the effect of NusA is increased in these conditions.

To assess the implication of the RNA hairpin in transcriptional pausing, we reasoned that mutants destabilizing the RNA hairpin should perturb the *hisL* pause half-life. Using this approach, we first tested a previously reported mutation [25]. This first mutation set (hisLS1) substitutes three residues in the 5’ side of the pause to destabilize the hairpin (Figure 1B). When performing transcription reactions, the *hisL* pause was found to be much less efficient (Figure 1C and 1D). When introducing mutations in the 3’ side of the stem to destabilize the hairpin (hisLS2) (Figure 1B), we also observed that the efficiency of the pause was severely diminished (Figure 1C and 1D). These results clearly show that, in our experimental conditions, the *hisL* pause is completely abolished when the hairpin structure is destabilized. Notably, these results suggest that transcriptional pausing is not efficiently achieved in these conditions even if the DNA sequence consensus required for pausing is present (Figure 1B). However, when both mutation sets are simultaneously introduced to re-establish base pair formation (hisLS3), the pause half-life is rescued to near wild-type level (Figure 1C and 1D). As previously observed, these results indicate that the structure of the RNA hairpin is important for the *hisL* pause half-life [25].

To assess the robustness of the mutational analysis, we performed a second round of mutations. In these experiments, 5’ (hisLS4) and 3’ (hisLS5) side mutations were introduced at the base of the RNA hairpin (Figure 1B). As expected, when performing transcription assays using the 5’ or 3’ mutant, the half-lives of the *hisL* pause could not be detected (Figure 1D). Importantly, when the two sets of mutations were simultaneously introduced (hisLS6), the pause half-life was not rescued (Figure 1D). This result either suggests that the RNA hairpin is not stably folded in this sequence context or that the introduced mutations perturb transcriptional pausing. The low efficiency of transcriptional pausing with the hisLS6 mutant is particularly intriguing since the predicted free energies of the WT and hisLS6 mutant hairpins are very similar (−5.1 and −5.5 kcal/mol, respectively). We next characterized the importance of the DNA consensus sequence for the *hisL* transcriptional pausing. When performing transcription reactions using a template containing a G to T mutation at the +1 position (Figure 1B, hisLS7), we found that the efficiency of the pause was abolished (Figure 1D), consistent with the DNA consensus sequence being highly important for this pause. When both the DNA consensus sequence and the 5’ side of the hairpin are mutated (hisLS8), a similar result was obtained (Figure 1D), as expected from the importance of both the hairpin and DNA consensus sequence. Thus, our data indicate that *hisL* transcriptional pausing is perturbed when either the RNA hairpin or the DNA consensus sequence is altered.

Together, in agreement with previous studies [25], our data show that transcriptional pausing at the *hisL* pause site is modulated by sequence changes within the hairpin structure (Figure 1D). It is striking that our mutational analysis yielded two very different results: while transcriptional pausing is rescued using the hisLS3 mutant, the mutant hisLS6 does not show significant pausing at the *hisL* pause site (Figure 1D). These results suggest that the identity of the hairpin residues is very important for *hisL* transcriptional pausing and that a mutagenesis approach may need additional data to infer the presence of a hairpin involved in the transcriptional process. For instance, NusA-dependent transcriptional pausing assays rely on the use of wild-type sequences and therefore provide an additional level of information regarding the potential role of a hairpin structure in pausing. As such, the use of both a mutagenesis approach and NusA to decipher the mechanism of transcriptional pausing is crucial to probe the formation and role of nascent RNA structures. For all the following investigated pause sites, we have employed an approach relying on the use of both hairpin mutants and NusA-dependent transcription reactions. Furthermore, in the light of our *hisL* data, in case the mutagenesis data yield inconclusive information about the presence of a hairpin structure, we will rely on the NusA-dependent assays to determine whether an RNA hairpin is involved in transcriptional pausing.

### Study of the well-known trpL pause site

We have characterized the *trpL* pause as it is one of the most studied pause sites along with *hisL* [18]. Similarly to *hisL*, the half-life of the *trpL* pause was previously shown to be modulated by both NusA and an RNA hairpin [18]. When performing in vitro transcription reactions, we observed that the *trpL* pause exhibits a half-life of 21.9 ± 1.6 s (Figure 1E and Supplementary Table S1). When repeating the same experiment in the presence of NusA, we found that the pause half-life was increased by ~2.4-fold (Figure 1E), consistent with the importance of the hairpin for transcriptional pausing. To characterize the importance of *trpL* hairpin for transcriptional pausing, we performed a mutagenesis analysis similar to what we performed for *hisL*. When disrupting the *trpL* hairpin by introducing mutations either in the 5’ side (trpLS1) or the 3’ side (trpLS2) of the hairpin, the half-life of the pause was completely abolished (Figure 1E). However, when simultaneously introducing both sets of mutations to re-establish base pairing interactions (trpLS3), the half-life of the *trpL* pause was not restore to levels observed with the wild-type sequence (Figure 1E). Similarly to what observed for the hisLS6 mutant (Figure 1D), the low efficiency of transcriptional pausing detected for the trpLS3 compensatory mutant suggests that the hairpin is either not stably formed or that transcriptional pausing is perturbed in this context. Nevertheless, the clear NusA effect on transcriptional pausing indicates that an RNA hairpin is involved in *trpL* transcriptional pausing and that sequence changes on either side of the structure strongly perturb pausing (Figure 1E).

### Study of the leuL pause site

**Figure 2.**
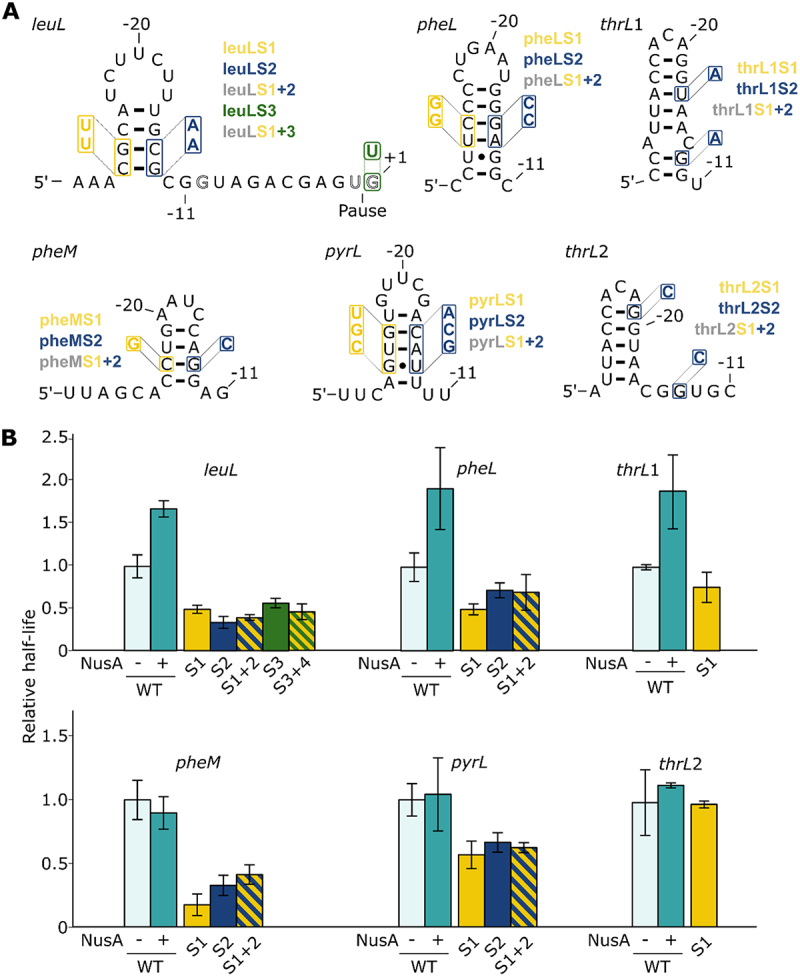
Study of pause sites *leuL, pheL, thrL1, thrL2, pheM* and *pyrL*. (A) Predicted structures of hairpins formed for the different pause regions. The mutants used in transcription assays are shown in each case. Additional mutations were performed in the DNA consensus sequence for the *leuL* pause. (B) Relative half-lives for the different pauses. The mutants are colour coded using the same nomenclature as shown in panel A. Hatched bars represent the combinations of the corresponding mutants. No combination of mutants was performed for thrL1 and thrL2 since the destabilization of the 5’ side of the hairpin (thrL1S1 and thrL2S1 mutants) did not significantly decrease the half-life of their respective pause.

The *leuL* pause was discovered in 1991 in *S*. Typhimurium and has been mapped near the end of the *leuL* leader peptide located upstream of the *leuA* operon (Figure 2A)[37]. Similarly to *hisL*, the *leuA* operon is regulated through transcription attenuation and *leuL* translation is sensitive to the concentration of charged levels of tRNA^Leu^ [37]. Although it was shown that the number of leucine codons affects transcription attenuation [37], no molecular mechanism was proposed to describe how transcriptional pausing controls gene expression. Consistent with the postulate of Landick and Yanofsky [7,44], the *leuL* pause was proposed to synchronize the processes of translation and transcription [37]. A small hairpin–the protector–was predicted upstream of the pause site and was suggested to induce pausing [37]. This hairpin is very similar in shape and position to the *hisL* hairpin. This pause site is also presenting the complete G_−10_Y_−1_G_+1_ consensus, suggesting that it may share a similar pausing mechanism than the *hisL* pause site.

We performed transcription assays using the *leuL* sequence and observed a pause site at the expected position, which is characterized by a half-life of 27.9 ± 3.8 s (Figure 2B and Supplementary Table S1). Bartkus et al. [37] reported that the *leuL* pause half-life is 4-fold higher (~1 min) than what we have obtained, suggesting that the pause efficiency is lower in our experimental conditions. To investigate the role of a putative RNA hairpin in *leuL* transcriptional pausing, we performed in vitro transcription assays in the presence of NusA. We observed that the pause half-life increased by ~1.7-fold (Figure 2B), suggesting a hairpin-stabilization effect by NusA on the paused elongation complex. Such an increase in pausing efficiency with NusA is very similar to what we have obtained for *hisL* and *trpL* (Figure 1D and 1E). Thus, these results suggest that transcription elongation within *leuL* produces a nascent RNA hairpin (Figure 2A) that is stabilized when bound to NusA, which ultimately leads to increased transcriptional pausing.

To investigate the influence of the hairpin structure on *leuL* transcriptional pausing, we predicted the structure upstream of the pause site in order to conduct a mutational analysis and obtained the exact same hairpin as in formerly predicted [47]. Therefore, we either allow or prevent the formation of the hairpin (Figure 2A). As expected, both 5’ and 3’ side mutants (leuLS1 and leuLS2) showed reduced half-lives by ~2.0-fold and ~2.9-fold, respectively (Figure 2B). However, the pause half-life in the compensatory mutant (leuLS3) was not increased compared to individual 5’ and 3’ mutants (Figure 2B), similarly to what we observed for hisL6 (Figure 1D) and trpL3 (Figure 1E) compensatory mutants.

Since the destabilization of the *leuL* hairpin did not completely abolish transcriptional pausing in the mutants leuLS1 and leuLS2 (Figure 2B), we reasoned that the residual transcriptional pausing could be due to the DNA consensus sequence. Indeed, it was previously shown that the consensus sequence is sufficient by itself to induce transcriptional pausing [30]. When introducing the G_+1_U mutation (leuLS4), we observed that the half-life decreased ~1.7-by fold compared to the wild-type sequence (Figure 2B). This effect is similar to what obtained with the leuLS1 and leuLS2 constructs containing mutations perturbing the formation of the hairpin structure. When mutating both the DNA consensus sequence and the 5’ side of the hairpin (leuLS5), we measured a similar decrease in half-life (~2.1-fold) than when mutating either region (Figure 2B). Thus, similarly to what obtained for *hisL* (Figure 1D), the half-life of the pause is not more severely affected when mutating both the DNA consensus sequence and the RNA structure. The residual pausing observed in this context is likely to be caused by either other consensus elements, such as G_−10_ and Y_−1_ and/or by an unexpected upstream RNA structure, that is, not disrupted in the 5’ mutant. However, it is clear that the importance of consensus is uneven for different pause sites, since the *hisL* pause is much less efficient when introducing the G_+1_ mutation (Figure 1D). Together, although compensatory mutations do not imply the presence of an RNA hairpin at the *leuL* pause site, a NusA-dependent increase of the pause half-life suggests that a nascent RNA hairpin structure may promote *leuL* transcriptional pausing.

### Study of pheL, thrL1, thrL2, pheM and pyrL pause sites

We expanded our study to five other pauses that were previously reported in a NET-Seq study [30], namely *pheL, thrL1, thrL2, pheM* and *pyrL*. The state of knowledge regarding these pauses is variable and deserves to be investigated. Together, even if these aforementioned pauses exhibit the suggested consensus sequence, we speculated that they could rely on a hairpin-dependent transcriptional pausing mechanism similar to the well-characterized *hisL* and *trpL* pause sites.

Secondary structure predictions suggested the presence of stem-loop structures for all analysed pause regions (Figure 2B). For all studied pause sites, although we observed structures similar to the *hisL* hairpin (Figure 1A), we obtained various predicted minimal-free energies for the RNA hairpins ranging from −2.2 to −7.8 kcal/mol, suggesting that these RNA structures may exhibit different properties to fold during transcription elongation. To assess their importance for pausing, transcription assays were performed for all pause sites with and without NusA, and a mutational analysis was also done to investigate the predicted structures.

Regarding the *pheL* pause, although transcription kinetics assays have been performed on this leader region and an RNA structure was predicted [38,39], we did not find any mention of transcriptional pausing beyond NET-Seq [30]. Our structure prediction led to an hairpin identical to the one previously predicted [38], even if it was not considered to be involved in transcriptional pausing. The *pheL* pause site exhibits similarities to *hisL, trpL* and *leuL* pauses. Indeed, it is located in the leader region of *pheA*, a gene involved in biosynthesis of both tyrosine and phenylalanine, and controls its expression by attenuation [39]. The half-life of the WT pause corresponds to 16.7 ± 2.9 s (Supplementary Table S1). Importantly, upon addition of NusA, the half-life of the pause was increased by ~1.9-fold (Figure 2B), suggesting the that the predicted hairpin is important for transcriptional pausing. When mutating either the 5’ or 3’ side of the predicted stem (pheLS1 and pheLS2 mutants), we found that the half-life was decreased by ~2.0-fold and 1.4-fold, respectively (Figure 2B). However, when inserting both 5’ and 3’ mutations to obtain a compensatory mutant (pheLS3), no clear compensation was obtained (Figure 2B). Thus, although the compensatory mutations did not give conclusive results, the clear effect obtained with NusA on the *pheL* pause half-life (Figure 2B) suggests the presence of a hairpin structure (Figure 2A) that is important for transcriptional pausing. The predicted *pheL* hairpin structure very likely corresponds to the ‘protector’ structure previously suggested [38] since both structures are highly similar.

For *thrL1* and *thrL2* pauses [40,42], there is little information available about their role. However, it was suggested that they could have a similar function in transcription regulation as *hisL*, that is regulating the termination frequency at attenuation sites [25]. Moreover, previous predictions suggested the presence of upstream RNA hairpins that could potentially be involved in the stabilization of the paused elongation complex [40,42]. The *thrL* gene encodes a leader peptide controlling the expression of the threonine biosynthetic operon *thrLABC*, which is related to the intracellular concentrations of threonine and isoleucine [48]. Both *thrL1* and *thrL2* pauses are found in this leader region, the latter being located 2 nt downstream. Therefore, they are sharing nearly the same predicted structure with an offset of 2 nt (Figure 2A). The two pause sites are the same as previously mapped and the structure is identical to the formerly predicted hairpin [40]. We built two constructs to study the influence of the structure on the two pauses independently. Experimentally, they both presented short but similar WT half-lives of, respectively, 7.5 ± 0.2 s and 7.7 ± 2.0 s (Supplementary Table S1). However, only *thrL1* responds to NusA resulting in a half-life increased by ~1.9-fold, suggesting that the completeness and the position of the hairpin is essential for NusA interaction. Both pauses with a 5’ mutation (thrL1S1, thrL2S1) lead to little or no decrease in their half-lives (Figure 2B), that is, respectively, ~1.3 and ~1.0-fold. Since these mutations are predicted to disrupt the structure (Supplementary Table S1, see ΔG values) but do not affect the pause half-life, the 3’ and compensatory mutants were not made. The pause-stabilizing interaction of NusA on *thrL1* strongly suggests that this pause, but not its 2 nt offset counterpart (*thrL2*), is stabilized by a cotranscriptionally folded RNA hairpin.

Both the *pyrL* and *pheM* genes are encoding for a leader peptide controlling the expression of respectively *pyrLBI* [49] and *pheMST* [50] operons by attenuation. For the *pyrL* pause [41], there is also little information that is known, but similarly to *thrL*, it was proposed to regulate transcription regulation similarly as *hisL* [25]. Predictions suggested the presence of upstream RNA hairpins potentially involved in the pause stabilization [40,42]. However, our assays revealed a pause site that is located ~24 nt upstream of the previously studied pause [41], thus our structural predictions gave a totally different hairpin structure. Finally, to our knowledge, very little is currently known about the structure and the role of *pheM* pause site and its sole existence is noticed [30], making our structural prediction the first ever discussed.

Our experiments showed that both pauses exhibit very similar behaviour regarding the impact of NusA and structural mutations on transcriptional pausing. The pause half-lives of the WT constructs for *pyrL* and *pheM* are 12.4 ± 1.6 s and 25.2 ± 3.9 s, respectively (Supplementary Table S1). When performing transcription assays in the presence of NusA, we did not obtain a significant difference on the pause half-life for both constructs (Figure 2B), suggesting that these pauses are not stabilized by NusA. In addition, the half-life of both pauses are decreased by ~1.5 to ~5.8-fold when 5’ or 3’ mutations are introduced (pyrLS1, pyrLS2, pheMS1 and pheMS2) (Figure 2B), suggesting that the *pyrL* and *pheM* sequences are important for transcriptional pausing. Similar effects were obtained when using the compensatory mutants (pyrLS3 and pheMS3) (Figure 2B), indicating that no rescue of pausing was obtained in these contexts. Thus, our results obtained for *pyrL* and *pheM* suggest that although the transcriptional pausing is affected by the RNA sequence, the presence of NusA does not affect the efficiency of pausing, which is different than what we observed for the other pause sites studied above.

### Pauses not regulated by nascent RNA hairpin

The *rfaQ* and *rnpB* pauses have previously been suggested to not be regulated by a nascent RNA hairpin structure [16,34–36]. While the *rfaQ* pause is found in the leader region of the lipopolysaccharide core heptosyltransferase 3 (*rfaQ*), the *rnpB* pause is the only one in our study that is not located in leader regions of biosynthetic operons, but is rather located in the coding region of the RNase P catalytic RNA component (*rnpB*). To validate the NusA effects that we have observed for *leuL, pheL* and *thrL1* (Figure 2B), we have performed transcription assays using *rfaQ* and *rnpB* as negative controls.

**Figure 3.**
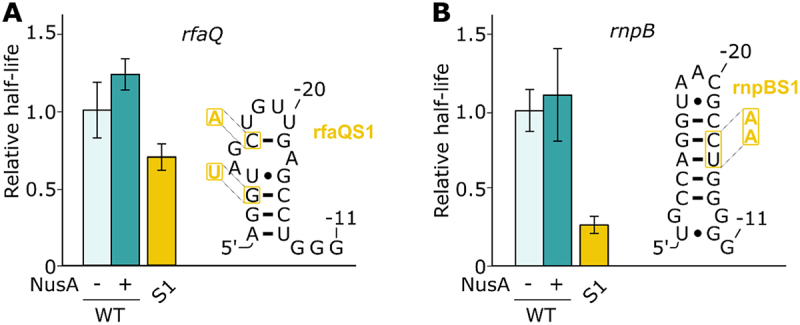
Study of the *rfaQ* and *rnpB* pause sites. (A and B) Relative pause half-lives of *rfaQ* (A) and *rfaQ* (B) pauses and the different mutants tested. The predicted structures of the respective hairpins are shown on the right. The different mutations used are shown on the structure. The mutants are depicted by their respective numbers (e.g. 1 for rfaQS1 or rnpBS1).

For both *rfaQ* and *rnpB*, transcription reactions revealed pause half-lives of 17.4 ± 3.1 s and 75.2 ± 10.1 s, respectively (Supplementary Table S1). As expected, NusA did not enhance transcriptional pausing significantly (Figure 3A and 3B), consistent with previous data suggesting that both *rfaQ* and *rnpB* do rely a hairpin-independent transcriptional pausing mechanism [16,34–36]. Furthermore, when altering the stability of the predicted *rfaQ* hairpin, the half-life was decreased by ~1.4-fold, indicating that the sequence is important for pausing (Figure 3A). Similarly, when analysing the *rnpB* pause site, we found that the destabilization of the hairpin led to a ~ 3.8-fold decrease of the pause half-life (Figure 3B). Therefore, in agreement with previous studies [16,34–36], our analysis of pause sites *rfaQ* and *rnpB* revealed that both pause sites do not rely on a NusA-dependent stabilization of an upstream RNA hairpin.

### Importance of consensus sequence and structures in high-throughput pausing datasets

**Figure 4.**
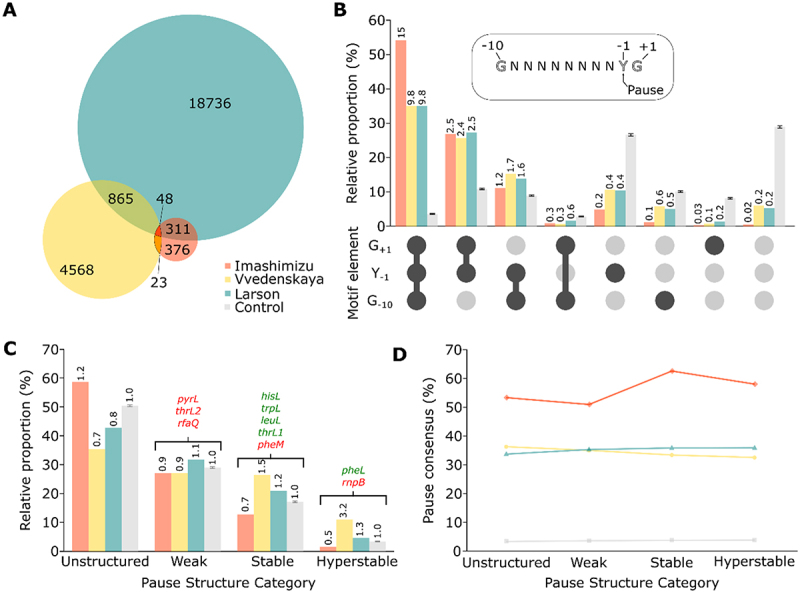
The three NET-Seq datasets are enriched for different characteristics. (A) Pauses overlap between datasets. The size of the circles is proportional to the number of identified pauses. The number of pauses identified in more than one study is indicated in the shared regions. The legend of the Control group is shown only for the colour scheme used in the other panels. (B) Frequency of all consensus variants among the three datasets. Numbers over the bars represent the fold change relative to the Control group. The residue Y in the consensus sequence denotes a pyrimidine. The datasets are identified using the same colour scheme as in panel A. (C) Distribution of predicted structures energy in all three sets, sorted in four energy bins (Unstructured: 0 to −1 kcal/mol, Weak: −1 to −3 kcal/mol, Stable: −3 to −6 kcal/mol, Hyperstable: −6 kcal/mol to lower energies). Numbers over the bars represent the fold change relative to the Control group. The datasets are identified using the same colour scheme as in panel A. Red and green pauses represent the presence or the absence of a NusA effect, respectively. (D) Distribution of consensus sequence for pausing among energy bins in the datasets. The datasets are identified using the same colour scheme as in panel A.

NET-Seq studies revealed thousands of pause sites in *E. coli*, indicating that transcriptional pausing is a widespread feature [30–32]. To investigate on a larger scale the role of putative RNA secondary structures in transcriptional pausing, we analysed the sequence upstream of reported pause sites. Interestingly, the three NET-Seq studies exhibited large variations in the number of pauses, namely Imashimizu [31] (n = 758), Larson [30] (n = 19 960) and Vvedenskaya [32] (n = 5 504). In addition, these studies did not consistently detect the same pause sites suggesting that different subsets of pauses were identified. For example, while the *hisL* pause was detected in the Larson study, it was not reported in the Imashimizu and Vvedenskaya studies. Such important variations could be due to pause calling stringency, resulting in more or less conservative pause annotations. Although the original data were produced using similar experimental procedures and strains (W3110 for Imashimizu and MG1655 for the two others), our analysis has raised considerably different characteristics. We found an unexpectedly low overlap between all26222pause sites, from which only 48 (<0.2%) are universally shared (Figure 4A and Supplementary Figure S3A). This shows that the datasets represent mostly different pauses, suggesting that a fraction of transcriptional pausing may occur stochastically.

All three studies independently found the same consensus sequence G_−10_Y_−1_G_+1_ that is at least partially responsible for pausing. Indeed, the consensus does not appear mandatory to induce pausing, since it is absent from a large portion of detected pauses in each study. Between 35% and 54% of pause sites exhibit the complete consensus sequence, which is ~10 to 15-fold more than the number expected from random sequences (Figure 4B, first group columns and Supplementary Figure S3B). The partial consensus Y_−1_G_+1_ was also considerably enriched ~2.5-fold compared to random sequences (Figure 4B, second group columns), suggesting that G_−10_ may not be as essential to induce pausing events. On the opposite, ~5% of the pauses from Larson and Vvedenskaya have none of the three consensus elements, while it is only 0.5% for Imashimizu (but ~30% of the random sequences) (Figure 4B, last group columns). The pauses presenting only a single consensus element or the G_−10_G_+1_ part are much less present, meaning that only these elements might not suffice to induce pausing (Figure 4B, fifth to seventh group columns). The great differences in frequencies suggest that the pauses population from Imashimizu rely more on the complete or partial consensus, while a wider proportion of pausing events from Larson and Vvedenskaya seemingly rely on other features, such as hairpin stabilization.

We then sorted the pauses based on the stability of RNA structures predicted to fold upstream of pause sites, which would contact the elongation complex similarly to *hisL*. A distance of 10 nt was used as a spacer between predicted structures and the pause sites to take into account the RNA:DNA hybrid. Based on the well-known *hisL* pause stabilizing hairpin as well as the other pause sites characterized above, we determined arbitrary thresholds and sorted the pauses in four categories. The first group contains unstructured pauses, meaning that no stable hairpin is predicted. The second group contains pauses preceded by weak structures, which are unlikely to stabilize paused elongation complexes (e.g. *pyrL, thrL2* and *rfaQ*). The third group contains pauses with stable structures, which could fold stably enough to interact with RNAP and NusA (e.g. *hisL, trpL, leuL, thrL1* and *pheM*). Finally, the last group is composed of hyperstable structures (e.g. *pheL* and *rnpB*). The classification of all the identified pauses shows that the ones from Imashimizu presented mostly (~58%) unstructured sequences, which represents a slight enrichment compared to random sequences (~1.2-fold) (Figure 4C and Supplementary Figure S3C). This is consistent with the results from the previous panel suggesting that most of the pause contained in this dataset would rely solely on consensus. On the opposite, the ones from Vvedenskaya are rather enriched for pauses containing stable and hyperstable structures (~1.5 and ~3.2-fold respectively).

We next investigated the relationship between the stability of the predicted RNA hairpin and pauses containing the complete or partial (Y_−1_G_+1_) consensus for each energy category. The proportion of pauses with consensus is strikingly even across the categories (Figure 4D and Supplementary Figure S3D). Overall, it suggests that there is no apparent relation between structure and consensus in these datasets, meaning that both features are neither exclusive nor related. Therefore, it remains hazardous to predict genuine hairpin-stabilization of transcriptional pauses based on NET-Seq results. However, we isolated a subgroup that could be targeted for further in vitro investigations, namely the pauses in each dataset presenting a stable predicted structure. Among these, we predicted 10 hairpin-stabilized pauses that exhibit identical properties (pause consensus sequence, RNA hairpin stability and the hairpin-pause site distance) to the *hisL* pause (Supplementary Figure S4). Interestingly, apart from *fimB* that is located within the 5’ UTR, all other pauses are found within coding regions, suggesting that they might be important to control transcription in a context of translating ribosomes. When performing a similar analysis in which more flexibility was allowed in the formation of the hairpin structure (see Materials and Methods for details), we retrieved a much larger number of pauses (561 candidates, see Supplementary File), suggesting that hairpin structures may modulate transcriptional pausing to a higher degree than previously thought. More work will be required to determine the exact molecular mechanisms involved in transcriptional pausing at these novel sites.

## Conclusion

We revealed three new interactions between NusA and previously mapped pause sites within *leuL, pheL* and *thrL1*, strongly suggesting that hairpin stabilizes the pause elongation complex similarly to the well-known *hisL* and *trpL* pauses. On the opposite, the pause sites of *pyrL, pheM* and *thrL2* are not relying on NusA activity. We also confirmed the absence of relation between both *rfaQ* and *rnpB* pauses and NusA-dependent hairpin stabilization. Although the use of NusA to reveal the role of hairpin structures in transcriptional pausing is valuable, it still requires further validation to clearly determine the molecular mechanism involved in pausing. For example, the effects of NusA on *leuL, pheL* and *thrL1* could result from NusA contacting the nascent transcript in a hairpin-independent manner via its RNA-binding domains. However, such a situation is less likely to occur in our analysis since our measurements specifically take into account the half-life of the paused transcript species (PT, 54 nt). Furthermore, even in the absence of a NusA effect, it is possible that NusA interacts with *pyrL, pheM* and *thrL2* transcripts, which could be explained if the corresponding hairpin structures are stably formed in the unbound state. Nevertheless, the presence of significant NusA effects – as observed here for *leuL, pheL* and *thrL1* – provides tantalizing evidence that nascent transcripts modulate transcriptional pausing.

From our analysis, when NusA is not involved in the transcriptional pausing process, it may be difficult to conclude the presence of an RNA hairpin using a mutagenesis approach since very few of our compensatory mutants yielded conclusive results. It is particularly striking that different sets of compensatory mutations in *hisL* showed distinctive pausing activities (e.g. hisLS3 vs hisLS6), thus suggesting that the formation of nascent hairpin structures is highly dependent on the sequence context. In agreement with this, a mutational analysis of 13 base pairs comprised within the *hisL* pause hairpin revealed that a compensatory effect was only clearly observed for three of them [25], indicating a low rate of base pair prediction (<25%). Therefore, it appears that the use of compensatory effects to determine the existence of an RNA hairpin may not be an efficient way to obtain a clear answer. This lack of clear compensatory effect is likely attributable to the formation of alternative secondary structures or to the presence of unstable RNA hairpin helices in the resulting mutants. Despite these limitations, we consider that mutational analysis are very powerful to ascertain the existence of RNA hairpins when compensatory effects can be observed. The use of additional mutations will be required to clearly ascertain the formation of RNA hairpin in the *leuL, pheL* and *thrL1* pause sites and to which degree they are involved in the stabilization of the elongating complexes. Our in vitro analysis inherently relies on the hypothesis that the studied hairpin structures exhibit similar structures as within their natural context. RNA structure predictions performed using the sequence from the natural context revealed very similar base pair probabilities when compared to the constructs used in our assays, except for *pheL* and *rnpB* (Supplementary Figure S5). However, when extending the upstream sequence for *pheL* and *rnpB*, we found that the predicted hairpin structures were observed (Supplementary Figure S6), suggesting that they are occurring in their natural context. Importantly, the use of RNA structure predictions only provides qualitative insights into the folding of the RNA since it does not take into account the cotranscriptional folding mechanism, which is likely to happen during transcription elongation.

Furthermore, our results obtained for the *hisL* and *leuL* pauses reveal that the DNA consensus sequence and the involved RNA hairpin need both to be functional for the transcriptional pausing to take place, indicating that the latter process may rely on multipartite determinants. The relationship between the consensus sequence and the RNA hairpin for transcriptional pausing is still unclear and will need further investigations. Also, it is still unknown if the pauses independent from NusA effect rely solely on the consensus sequence or on additional features, such as alternative secondary structures or sequence motifs located outside of the DNA-RNA hybrid.

The NET-Seq data show that 2125 pauses located across *E. coli* are occurring without a partial or complete consensus sequence, but present a stable structure and are therefore likely to depend solely on the stabilization of a hairpin structure. Additionally, 3602 pause sites that exhibit both an RNA hairpin and the consensus sequence are likely to rely on both features for pausing, suggesting that RNA hairpins may be widely spread across pause sites. Massive amount of in vitro validation is required and therefore an automated method needs to be developed. These results brought us to conclude that we only understand a handful of pause sites thoroughly and the genome-wide pausing landscape is still poorly documented.

## Materials and methods

### Template design

Templates used or in vitro transcription reactions were obtained through PCR reactions (Supplementary Table S2) using DNA oligonucleotides (Integrated DNA Technologies) (Supplementary Table S3). For all constructs, the template was used and only the sequence corresponding to the pause varied. The template consists of the *lacUV5* promoter fused to a 14 nt initiation sequence, which is followed by the pause-specific genomic region corresponding to positions −30 to + 5 relative to the pause site. This region was selected to contain any putative RNA hairpin and the DNA consensus sequence. The sequence added after the pause site allows to sufficient resolution between transcripts corresponding to the pause (54 nt) and full-length (77 nt) products. The rationale to engineer the 5’ and 3’ mutants was based on secondary structure predictions in which the minimum number of residues was changed to alter the predicted hairpin structure. The selected 5’ and 3’ mutants were predicted to allow the formation of a hairpin similar to the WT structure.

### RNA structure predictions

Using the *hisL* pause site as a model, we postulated that RNA structures involved in transcriptional pausing would likely be located between positions −30 to −11 relatively to the pause site. The downstream sequence of the pause site (−10 to −1) was omitted to exclude the RNA:DNA hybrid region, which is not available for folding. We also excluded the region −54 to −35 since it is expected to form a stem-loop structure not interfering with the downstream sequence. The analysis of the natural sequence was done using a transcript length similar to the one used in our in vitro assays or using an extended sequence (*pheL* and *rnpB*). The prediction of RNA structures was determined using a local version of RNAfold (ViennaRNA suite 2.4.11 [51]) with default parameters. The software produced a dot-bracket representation of the predicted structure and the associated minimal-free energy (MFE) or ΔG. The dot-bracket output was then used with R2R [52] to visualize secondary structures. The obtained predictions reflect the most stable structures and therefore the most likely to interact with the RNA polymerase when located at the pause site.

### Single-round in vitro transcription assays

All transcription reactions were performed in 20 mM Tris-HCl pH 8.0, 100 mM KCl, and 20 mM MgCl_2_. To obtain the transcription complex, the DNA template (450 nM) was incubated for 5 min at 37°C with the *E. coli* RNAP (900 nM) and σ70 factor (1.4 µM). To initiate transcription elongation, a GUU trinucleotide (10 µM), ATP (2.5 µM), GTP (2.5 µM), and [α-^32^P] UTP (11 kBq/µL) were added and incubated 10 min at 37°C. Samples were then diluted twice in buffer and were filtered through Sephadex G-50 columns (Sigma-Aldrich). Complete elongation of transcripts was started by the addition of all NTP (25 µM) and heparin (0.9 µg/µL) to allow single round conditions. NusA transcription factor (75 nM) was added to the reaction when required. For each timepoint, a aliquot was taken and the reaction was stopped in chilled formamide (60%). Transcription products were resolved on 8% polyacrylamide gels and quantified using QuantityOne software (Bio-Rad).

### Determination of transcriptional pause half-life

To monitor transcriptional pausing, we quantified the signal intensity in a rectangle area corresponding to the paused transcript (PT, 54 nt) and including the pause escaped transcripts (from PT, 54 nt to FL, 77 nt) over time using the QuantityOne software (Bio-Rad). Thereafter, the pause half-life was obtained by fitting the proportion of paused transcript over the escaped transcripts to an exponential decay model using the R library nlstools (version 1.0.2) [53]. Half-lives fold change was evaluated between each condition and the WT values, and the statistical significance was evaluated with Welch’s *t*-test [54]. We considered a clear effect on half-life with *p*-value below 0.05. Each pause site was analysed at least twice. A sequencing ladder was used to precisely assess the position of the paused transcript. Sample series handling and treatments were managed using homemade Python 3.8.5 scripts (available upon request).

### High-throughput pausing dataset characterization

Considering that pause coordinates of two of the three datasets were provided using the MG1655 reference genome (Larson and Vvedenskaya), the pause sequences from position −49 to +1 were extracted from the W3110 reference genome for Imashimizu, and aligned on the MG1655 (NC_000913.3) genome to compare pause positions between the three different datasets (Figure 4A). We also did a sensitivity analysis, where an offset of at most 4 nt was used to match pause positions between datasets (Supplementary Figure S3A). Overlaps were represented in a Venn diagram generated with BioVenns [55].

For every pause site, the RNA structure was predicted using the sequence from positions −30 and −11 upstream of each pause site. The distribution of ΔG was compared to a group of 100 control datasets, each consisting of26222random MG1655 genomic sequences. The size of the control datasets was chosen considering the combined sizes of the three sample datasets. Predicted RNA structures characterized by energies falling between 0 and −1 kcal/mol were assigned to an unstructured group. The second group (−1 to −3 kcal/mol) was assigned to the presence of weak structures. The third group (−3 to −6 kcal/mol) was assigned to the presence of stable structures. Finally, the last group (−6 kcal/mol to lower energies) was assigned to hyperstable structures.

The identification of transcriptional pauses within the third group (Supplementary Figure S4) was performed using criteria matching the *hisL* pause. For instance, the screen was done to identify pauses having the consensus sequence, an RNA stem of 5 bp, a loop of 8 nt and a distance of 10 nt between the stem and the pause site.

## Supplementary Material

Supplemental Material

## Data sharing statement

Quantified in vitro results presented here are available in Supplementary Table S1. NET-Seq data are available as published by their respective authors (GSE62102, GSE56720 and SRP071742). The scripts that were used to analyze the NET-Seq data used in our study are available upon request to the authors.
