# Supplementary material for: Investigating the role of RNA structures in transcriptional pausing using in vitro assays and in silico analyses
Source: RNA Biol. 2022 Jul 14;19(1):916–27. doi: 10.1080/15476286.2022.2096794 (PMC9291695; doi:10.1080/15476286.2022.2096794)
Supplement: Supplemental Material [file KRNB_A_2096794_SM3212.zip › Jeanneau_RNABiol_2022_FigSupp revised.docx]

Investigating the role of RNA structures in transcriptional pausing using *in vitro* assays and *in silico* analyses

**Simon Jeanneau^1^, Pierre-Étienne Jacques^*1,2^ and D.A. Lafontaine^*1^**

^1^Department of Biology, Faculty of Science, Université de Sherbrooke, Sherbrooke, Quebec, Canada, J1K 2R1.

^2^Centre de Recherche du CHUS, Université de Sherbrooke, Sherbrooke, Quebec, Canada, J1H 5N4.

*To whom correspondence should be addressed: Pierre-Etienne.Jacques@usherbrooke.ca and Daniel.Lafontaine@usherbrooke.ca


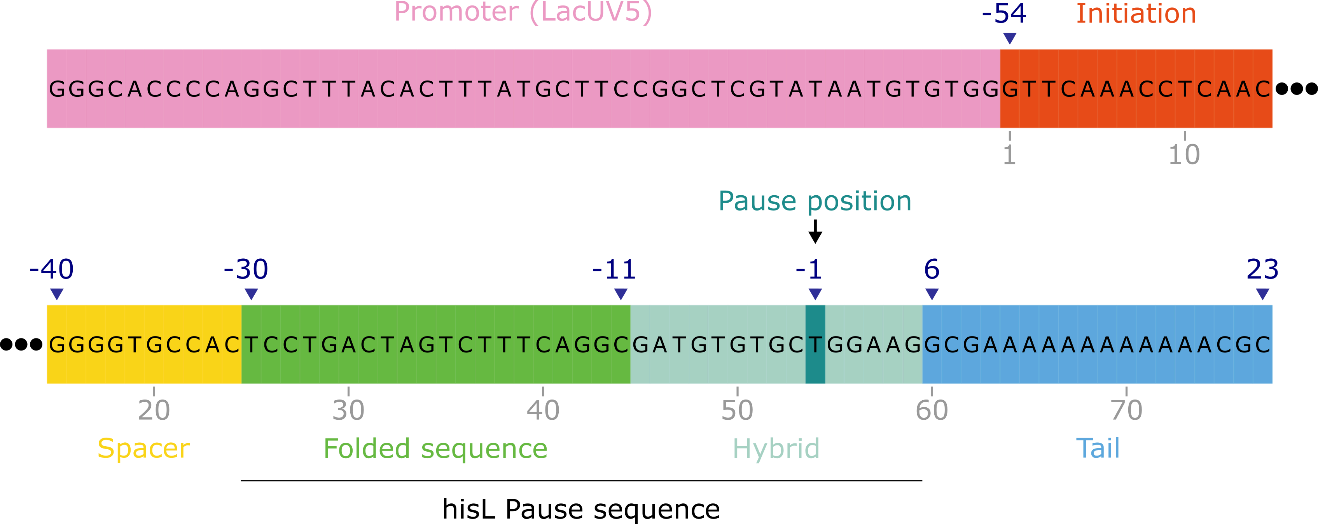


**Supplementary Figure S1. Schematic of the *in vitro* transcription template.** The sequence of the *hisL* pause was used as an example. The colors depict the different regions of the template. The upper numbering denotes the positions relatively to the pause site. The lower numbering shows the positions relatively to the beginning of the *lacUV5* promoter sequence.


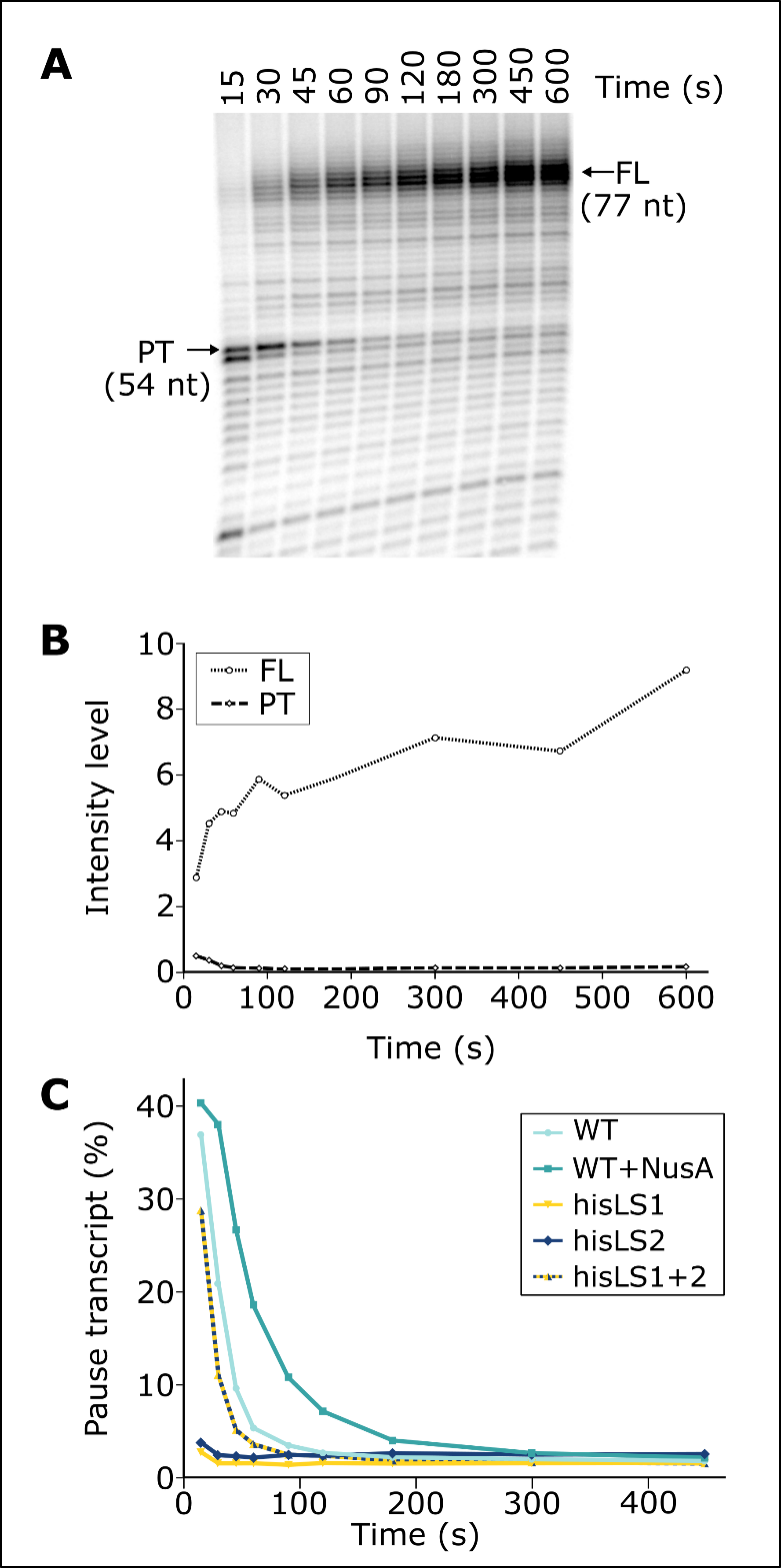


**Supplementary Figure S2. Transcription assays and analysis of *hisL* pause half-life.** (A) Extended image of the gel presented in Figure 1C (Full-length transcript (FL) and Paused transcript (PT). (B) Intensity levels of FL and PT bands over the time reaction. Data points were connected with lines. (C) The ratio of the pause transcript over time follows an exponential decay. Data for the WT in the presence and absence of NusA, and studied mutants, are shown. The data are fitted with an exponential decay and the obtained half-life values are presented in the Supplementary Table S1.


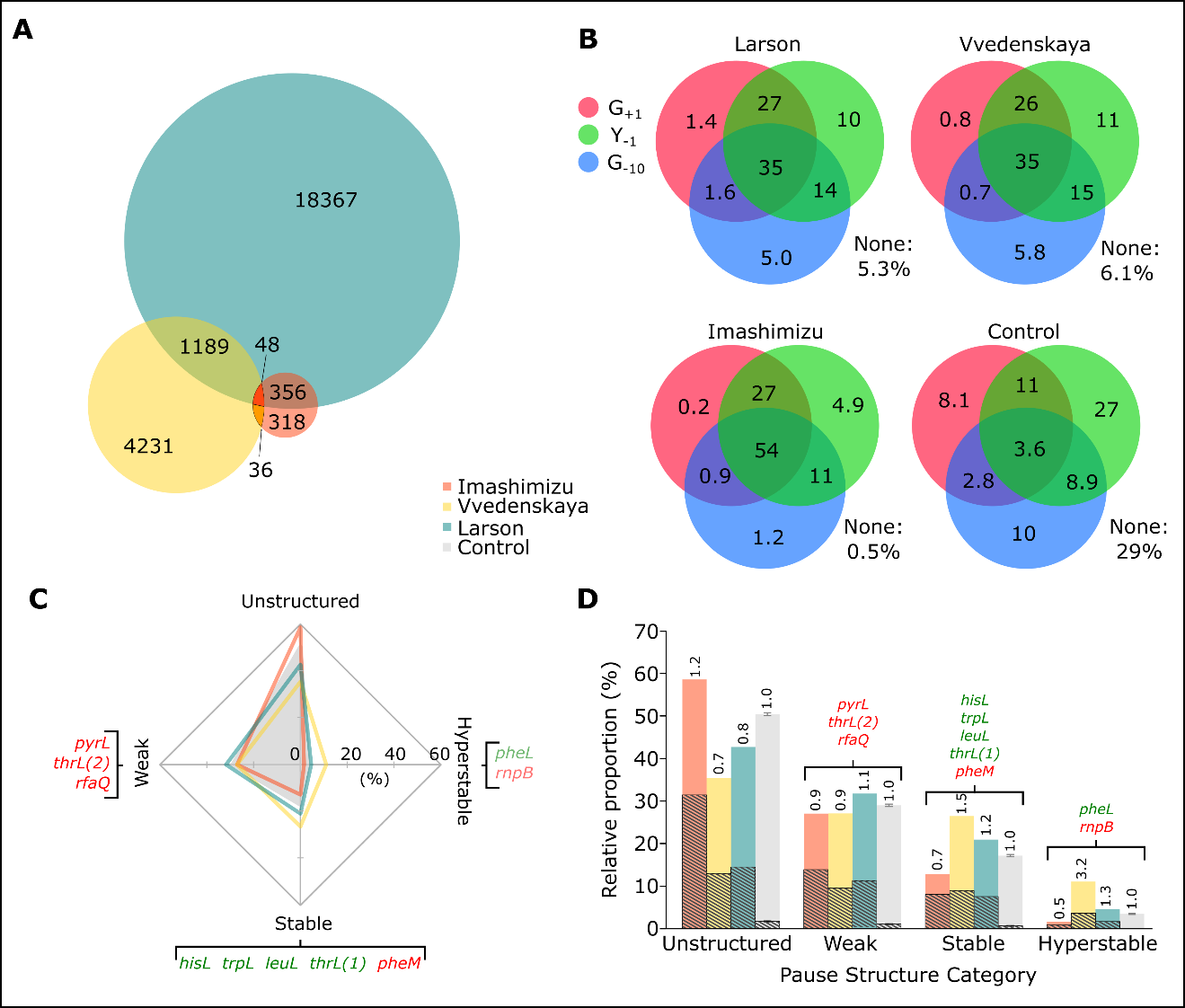


**Supplementary Figure S3. Bioinformatic analysis of transcriptional pauses identified in three different NET-Seq studies.** (A) Overlap of the three datasets after sensitivity analysis using more permissive parameters (see Methods). The size of the circles is proportional to the number of identified pauses. The number of pauses identified in more than one study are indicated in the shared regions. (B) Alternative representation of Figure 4B in which is shown the proportion of consensus elements among each dataset using simple non proportional pie charts. (C) Representation of the three datasets using a web chart to monitor the global distribution of the predicted energy. Red and green pauses represent the presence or the absence of a NusA effect, respectively. (D) Relative proportion of the identified pauses across the predicted energies. The proportion of pauses are shown in the presence (meshed) or absence (non-meshed) of the consensus sequence. Red and green pauses represent the presence or the absence of a NusA effect, respectively.


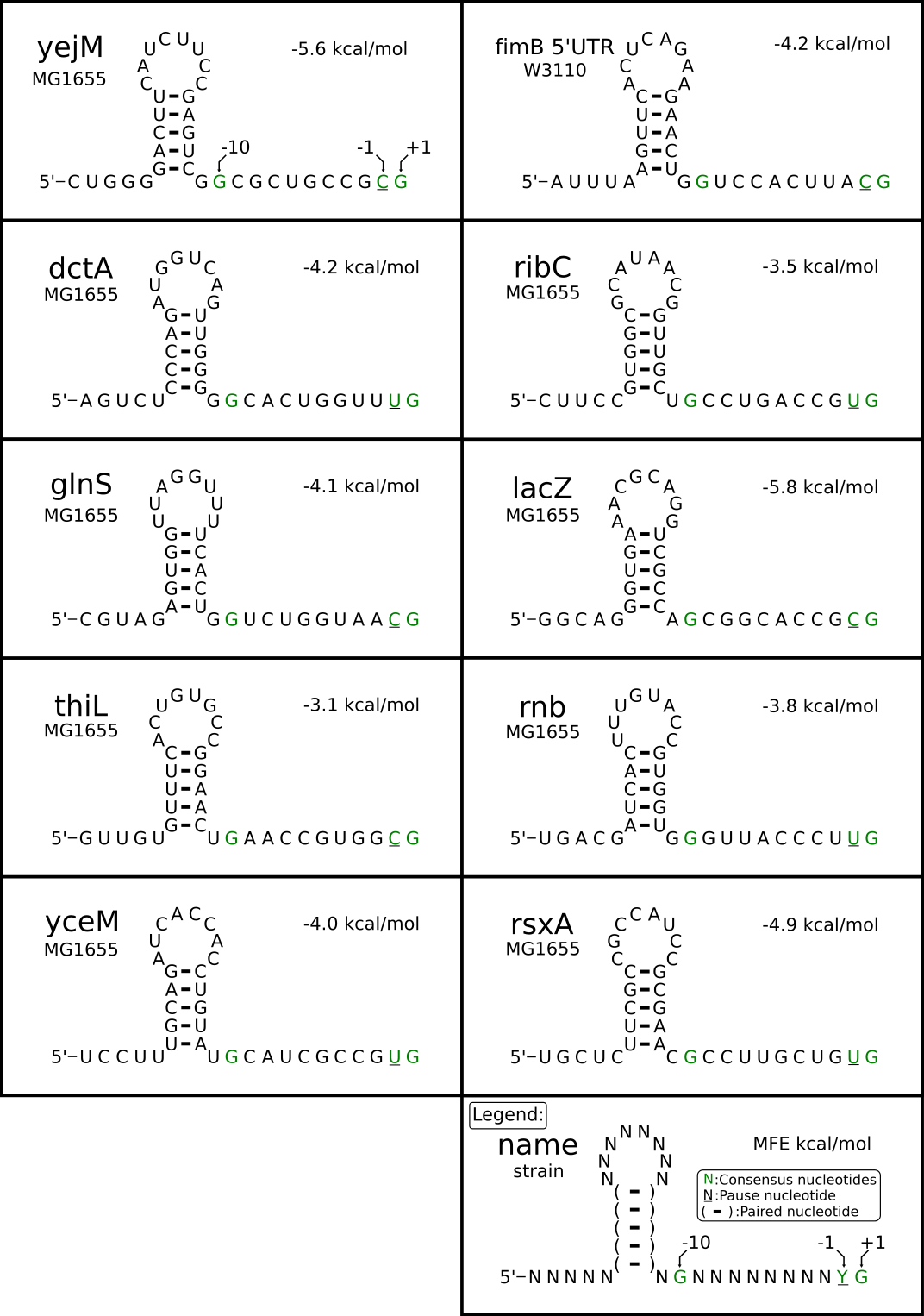
**Supplementary Figure S4. Transcriptional pauses identified as potential candidates to be modulated by the formation of hairpin structures.** Transcriptional pauses with predicted structures, neighboring energies (MFE) and complete consensus sequence that are similar to the *hisL* pause site. The bottom right panel represents the legend of the motifs. While the *fimB* structure is located in the 5' UTR domain, all other structures are found within coding regions.

**
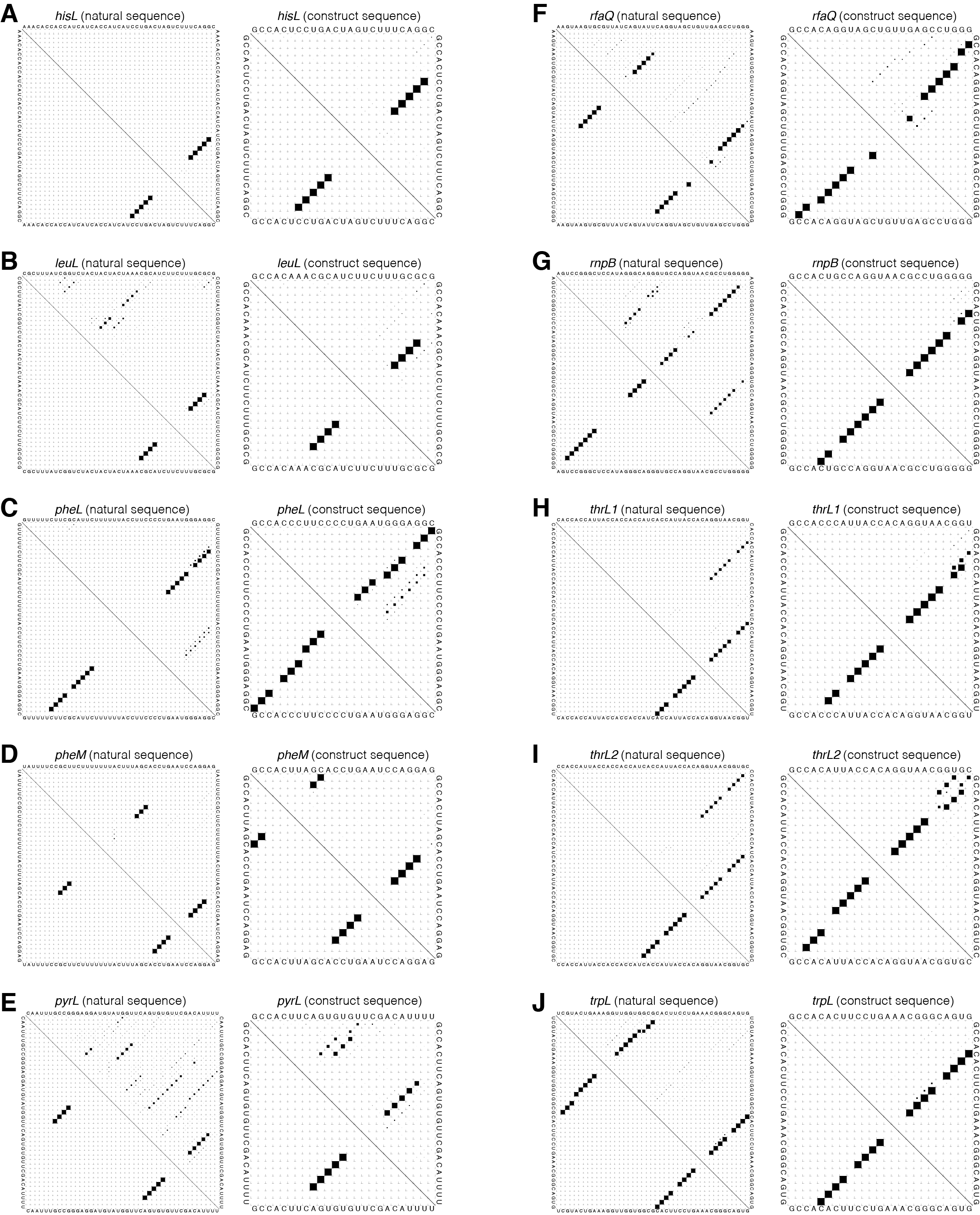
**

**Supplementary Figure S5. Pairing probability matrices of the structural landscape for each transcriptional pauses in natural and *in vitro* contexts.** For each pause site, the left-sided matrix shows the structural landscape of the pause in the natural context (i.e., the wild-type genomic sequence from position -54 to -10). The right-sided matrix shows the structural landscape in *in vitro* constructs (i.e., the construct sequence from position -35 to -10). The bottom-left side of each matrix represents the most probable structure of the population where dark squares show a base pair between nucleotides depicted on both axis. The top-right side of the matrix displays all probable base pairs within the structural ensemble, including the pairs forming the most probable structure. The size of the dark squares shows the relative probability of a base pair between the corresponding nucleotides on each axis.

**
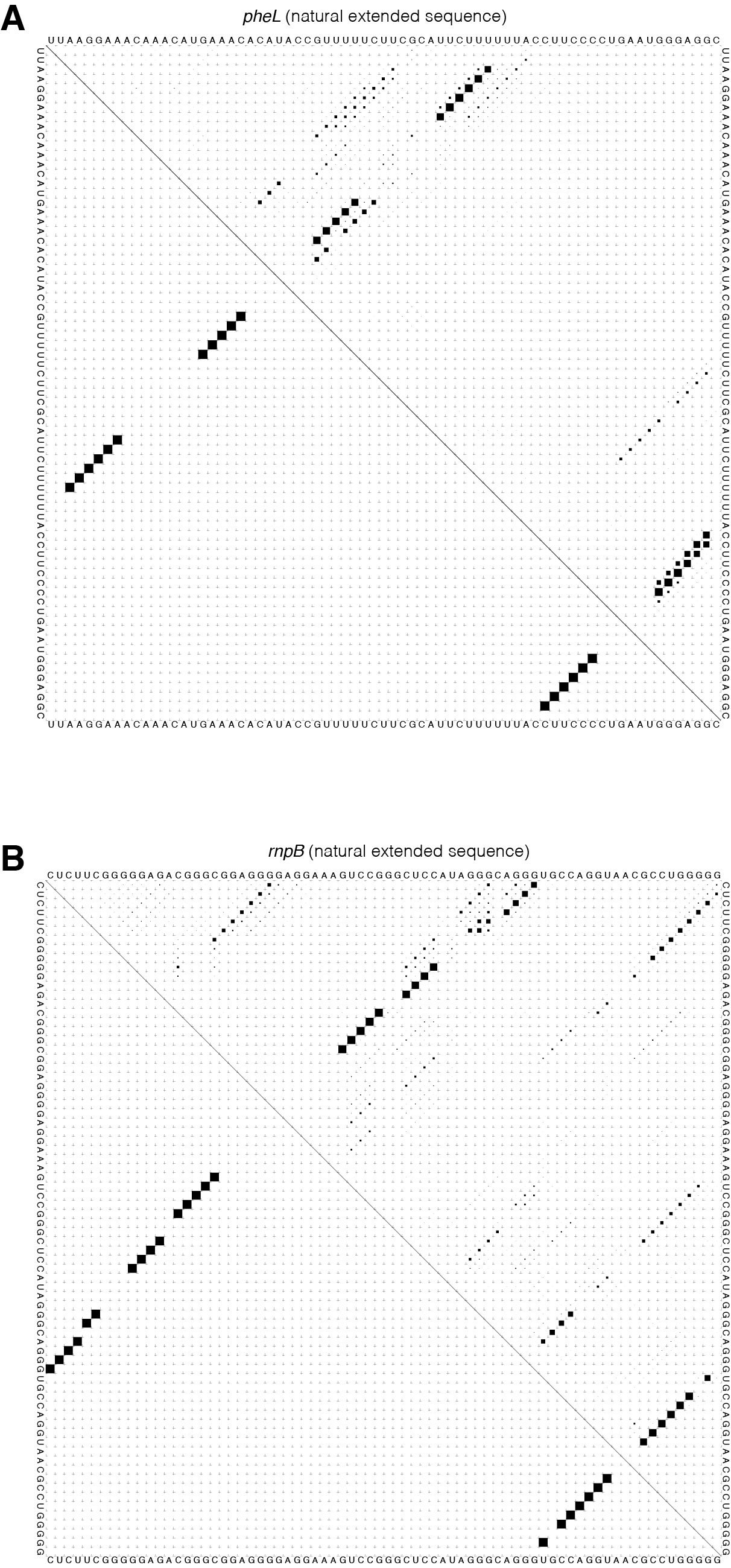
**

**Supplementary Figure S6. Pairing probability matrices of the structural landscape for *pheL* and *rnpB* transcriptional pauses in an extended natural context.** The bottom-left side of each matrix represents the most probable structure of the population where dark squares show a base pair between nucleotides depicted on both axis. The top-right side of the matrix displays all probable base pairs within the structural ensemble, including the pairs forming the most probable structure. The size of the dark squares shows the relative probability of a base pair between the corresponding nucleotides on each axis. Both (A) *pheL* and (B) *rnpB* sequences contain elements likely to fold with upstream sequence from greater range than previous predictions.

**Supplementary Table S1.** **Parameters determined using *in vitro* transcriptional pausing assays**. For each template studied, predicted structure stabilities, half-lives, number of replicates and standard deviation are shown. The fold change and p-value refer to the difference from the WT half-life value. n.a. (not applicable) refers to nonexistent values and n.d. (not detected) refers to the measured value below the detection limit.

| **Template** | | **ΔG (kcal/mol)** | **Half-life (s)** | **Number of replicates** | **SD (s)** | **Fold change** | **Fold change p-value** |
| --- | --- | --- | --- | --- | --- | --- | --- |
| hisL | WT | -5.1 | 8.5 | 9 | 1.4 | n.a. | n.a. |
|  | +NusA | -5.1 | 17.3 | 3 | 2.0 | 2.0 | 8.7E-03 |
|  | S1 | -0.4 | n.d. | 3 | n.d. | n.d. | n.a. |
|  | S2 | 0.0 | n.d. | 3 | n.d. | n.d. | n.a. |
|  | S1+2 | 0.0 | 9.1 | 3 | 0.6 | 1.1 | 9.2E-01 |
|  | S3 | -0.4 | n.d. | 3 | n.d. | n.d. | n.a. |
|  | S4 | 0.0 | n.d. | 3 | n.d. | n.d. | n.a. |
|  | S3+4 | -5.5 | n.d. | 3 | n.d. | n.d. | n.a. |
|  | S5 | -5.1 | n.d. | 3 | n.d. | n.d. | n.a. |
|  | S1+5 | -0.4 | n.d. | 3 | n.d. | n.d. | n.a. |
| trpL | WT | -4.7 | 21.9 | 5 | 1.6 | n.a. | n.a. |
|  | +NusA | -4.7 | 52.1 | 5 | 7.1 | 2.4 | 6.3E-03 |
|  | S1 | -3.0 | n.d. | 5 | n.d. | n.d. | n.a. |
|  | S2 | 0.0 | n.d. | 3 | n.d. | n.d. | n.a. |
|  | S1+2 | -3.1 | n.d. | 4 | n.d. | n.d. | n.a. |
| leuL | WT | -4.2 | 27.9 | 5 | 3.8 | 1.0 | n.a. |
|  | +NusA | -4.2 | 47.0 | 3 | 2.7 | 1.7 | 2.5E-04 |
|  | S1 | 0.0 | 14.1 | 3 | 1.4 | 2.0 | 4.9E-04 |
|  | S2 | 0.0 | 9.6 | 3 | 2.0 | 2.9 | 1.2E-04 |
|  | S1+2 | -0.7 | 11.3 | 4 | 1.0 | 2.5 | 3.5E-04 |
|  | S3 | -4.2 | 16.3 | 3 | 1.6 | 1.7 | 1.1E-03 |
|  | S1+3 | 0.0 | 13.2 | 3 | 2.7 | 2.1 | 8.9E-04 |
| pheL | WT | -7.8 | 16.7 | 5 | 2.9 | n.a. | n.a. |
|  | +NusA | -7.8 | 32.5 | 7 | 8.2 | 1.9 | 3.9E-03 |
|  | S1 | -5.5 | 8.3 | 3 | 1.1 | 2.0 | 1.4E-03 |
|  | S2 | -0.7 | 12.1 | 3 | 1.5 | 1.4 | 2.4E-02 |
|  | S1+2 | -9.6 | 11.7 | 3 | 3.6 | 1.4 | 1.1E-01 |
| pheM | WT | -4.5 | 25.2 | 3 | 3.9 | n.a. | n.a. |
|  | +NusA | -4.5 | 22.6 | 4 | 3.2 | 1.1 | 4.0E-01 |
|  | S1 | -0.5 | 4.4 | 3 | 2.1 | 5.8 | 3.5E-03 |
|  | S2 | 0.0 | 8.2 | 3 | 2.0 | 3.1 | 6.9E-03 |
|  | S1+2 | -3.7 | 10.4 | 3 | 1.9 | 2.4 | 1.0E-02 |
| pyrL | WT | -2.2 | 12.4 | 4 | 1.6 | n.a. | n.a. |
|  | +NusA | -2.2 | 12.9 | 3 | 3.6 | 1 | 8.3E-01 |
|  | S1 | 0.0 | 7.0 | 3 | 1.3 | 1.8 | 5.1E-03 |
|  | S2 | 0.0 | 8.3 | 3 | 1.0 | 1.5 | 8.0E-03 |
|  | S1+2 | -3.6 | 7.8 | 4 | 0.5 | 1.6 | 6.8E-03 |

**Supplementary Table S1** **In vitro half-lives of transcriptional pauses (continued).**

| **Template** | | **ΔG (kcal/mol)** | **Half-life (s)** | **Number of replicates** | **SD (s)** | **Fold change** | **Fold change p-value** |
| --- | --- | --- | --- | --- | --- | --- | --- |
| thrL1 | WT | -5.0 | 7.5 | 3 | 0.2 | n.a. | n.a. |
|  | +NusA | -5.0 | 14.4 | 3 | 3.3 | 1.9 | 7.0E-02 |
|  | S1 | 0.0 | 5.7 | 3 | 1.4 | 1.3 | 1.4E-01 |
| thrL2 | WT | -2.4 | 7.7 | 3 | 2.0 | n.a. | n.a. |
|  | +NusA | -2.4 | 8.8 | 2 | 0.2 | 1.1 | 4.6E-01 |
|  | S1 | -1.2 | 7.6 | 2 | 0.2 | 1.0 | 9.3E-01 |
| rfaQ | WT | -2.5 | 17.4 | 3 | 3.1 | n.a. | n.a. |
|  | +NusA | -2.5 | 21.4 | 3 | 1.8 | 1.2 | 1.4E-01 |
|  | S1 | 0.0 | 12.1 | 3 | 1.5 | 1.4 | 8.1E-02 |
| rnpB | WT | -9.1 | 75.2 | 3 | 10.1 | n.a. | n.a. |
|  | +NusA | -9.1 | 82.8 | 3 | 22.4 | 1.1 | 6.3E-01 |
|  | S1 | -1.4 | 19.7 | 3 | 4.2 | 3.8 | 4.9E-03 |

**Supplementary Table S2. PCR constructs used for *in vitro* transcription assays.**

| **Constructs** |  | **Oligonucleotides** |
| --- | --- | --- |
| hisL WT | | 2223JFN-3763SJ |
| hisLS1 | | 2223JFN-3764SJ |
| hisLS2 | | 2223JFN-4071SJ |
| hisLS1+2 | | 2223JFN-4072SJ |
| hisLS3 | | 2223JFN-4068SJ |
| hisLS4 | | 2223JFN-4104SJ |
| hisLS3+4 | | 2223JFN-4069SJ |
| hisLS5 | | 2223JFN-4101SJ |
| hisLS1+5 | | 2223JFN-4100SJ |
| trpL WT | | 2223JFN-3947SJ |
| trpLS1 | | 2223JFN-4056SJ |
| trpLS2 | | 2223JFN-3975SJ |
| trpLS1+2 | | 2223JFN-4057SJ |
| rnpB WT | | 2223JFN-3950SJ |
| rnpBS1 | | 2223JFN-3978SJ |
| rfaQ WT | | 2223JFN-3951SJ |
| rfaQS1 | | 2223JFN-4066SJ |
| leuL WT | | 2223JFN-3943SJ |
| leuLS1 | | 2223JFN-3971SJ |
| leuLS2 | | 2223JFN-4050SJ |
| leuLS1+2 | | 2223JFN-4051SJ |
| leuLS3 | | 2223JFN-4103SJ |
| leuLS1+3 | | 2223JFN-4102SJ |
| pheL WT | | 2223JFN-3949SJ |
| pheLS1 | | 2223JFN-4060SJ |
| pheLS2 | | 2223JFN-3977SJ |
| pheLS1+2 | | 2223JFN-4061SJ |
| thrL1 WT | | 2223JFN-3946SJ |
| thrL1S1 | | 2223JFN-3974SJ |
| pheM WT | | 2223JFN-3948SJ |
| pheMS1 | | 2223JFN-4058SJ |
| pheMS2 | | 2223JFN-3976SJ |
| pheMS1+2 | | 2223JFN-4059SJ |
| pyrL WT | | 2223JFN-3945SJ |
| pyrLS1 | | 2223JFN-4054SJ |
| pyrLS2 | | 2223JFN-3973SJ |
| pyrLS1+2 | | 2223JFN-4055SJ |
| thrL2 | | 2223JFN-3955SJ |
| thrL2S1 | | 2223JFN-3980SJ |
|  | |  |

**Supplementary Table S3. Summary of oligonucleotides used in this study.**

| **Oligonucleotides** | **Sequences 5'-3'** | |
| --- | --- | --- |
| 2223JFN | GGGCACCCCAGGCTTTACACTTTATGCTTCCGGCTCGTATAATGTGTGGGTTCAAACCTCAACGGGGTGCCAC |  |
| 3763SJ | GCGTTTTTTTTTTTTCGCCTTCCAGCACACATCGCCTGAAAGACTAGTCAGGAGTGGCACCCCGTTGAGGTTTGAACCC |  |
| 3764SJ | GCGTTTTTTTTTTTTCGCCTTCCAGCACACATCGCCTGAAAGACTAGTTAATAGTGGCACCCCGTTGAGGTTTGAACCC |  |
| 3943SJ | GCGTTTTTTTTTTTTCGCCGCTCACTCGTCTACCGCGCAAAGAAGATGCGTTTGTGGCACCCCGTTGAGGTTTGAACCC |  |
| 3945SJ | GCGTTTTTTTTTTTTCGCCAGACGCGGTAAGACAAAATGTCGAACACACTGAAGTGGCACCCCGTTGAGGTTTGAACCC |  |
| 3946SJ | GCGTTTTTTTTTTTTCGCTACGCGTCAGCCCGCACCGTTACCTGTGGTAATGGGTGGCACCCCGTTGAGGTTTGAACCC |  |
| 3947SJ | GCGTTTTTTTTTTTTCGCTACGCATGGTGAATACACTGCCCGTTTCAGGAAGTGTGGCACCCCGTTGAGGTTTGAACCC |  |
| 3948SJ | GCGTTTTTTTTTTTTCGCTTCTCACGCGCTAGCCTCCTGGATTCAGGTGCTAAGTGGCACCCCGTTGAGGTTTGAACCC |  |
| 3949SJ | GCGTTTTTTTTTTTTCGCTTCACACGACGAAACGCCTCCCATTCAGGGGAAGGGTGGCACCCCGTTGAGGTTTGAACCC |  |
| 3950SJ | GCGTTTTTTTTTTTTCGCTGGTCGTGGGTTTCCCCCCCAGGCGTTACCTGGCAGTGGCACCCCGTTGAGGTTTGAACCC |  |
| 3951SJ | GCGTTTTTTTTTTTTCGCAAAGCACGCTACCGCCCCAGGCTCAACAGCTACCTGTGGCACCCCGTTGAGGTTTGAACCC |  |
| 3955SJ | GCGTTTTTTTTTTTTCGCTGTACGCGTCAGCCCGCACCGTTACCTGTGGTAAGTGGCACCCCGTTGAGGTTTGAACCC |  |
| 3971SJ | GCGTTTTTTTTTTTTCGCCGCTCACTCGTCTACCGTTCAAAGAAGATGCGTTTGTGGCACCCCGTTGAGGTTTGAACCC |  |
| 3973SJ | GCGTTTTTTTTTTTTCGCCAGACGCGGTAAGACAAACGTTCGAACACACTGAAGTGGCACCCCGTTGAGGTTTGAACCC |  |
| 3974SJ | GCGTTTTTTTTTTTTCGCTACGCGTCAGCCCGCACTGTTTCCTGTGGTAATGGGTGGCACCCCGTTGAGGTTTGAACCC |  |
| 3975SJ | GCGTTTTTTTTTTTTCGCACTTCCTGAAACGGGCACTGGCCGTTTCATGAAGTGTGGCACCCCGTTGAGGTTTGAACCC |  |
| 3976SJ | GCGTTTTTTTTTTTTCGCTTCTCACGCGCTAGCCTCGTGGATTCAGGTGCTAAGTGGCACCCCGTTGAGGTTTGAACCC |  |
| 3977SJ | GCGTTTTTTTTTTTTCGCTTCACACGACGAAACGCCGGCCATTCAGGGGAAGGGTGGCACCCCGTTGAGGTTTGAACCC |  |
| 3978SJ | GCGTTTTTTTTTTTTCGCTGGTCGTGGGTTTCCCCCCCTTGCGTTACCTGGCAGTGGCACCCCGTTGAGGTTTGAACCC |  |
| 3980SJ | GCGTTTTTTTTTTTTCGCTGTACGCGTCAGCCCGCAGCGTTACGTGTGGTAAGTGGCACCCCGTTGAGGTTTGAACCC |  |
| 4050SJ | GCGTTTTTTTTTTTTCGCCGCTCACTCGTCTACCGCGCAAAGAAGATGAATTTGTGGCACCCCGTTGAGGTTTGAACCC |  |

**Supplementary Table S3. Summary of oligonucleotides used in this study (continued).**

| Oligonucleotides | Sequences 5'-3' |
| --- | --- |
| 4051SJ | GCGTTTTTTTTTTTTCGCCGCTCACTCGTCTACCGTTCAAAGAAGATGAATTTGTGGCACCCCGTTGAGGTTTGAACCC |
| 4054SJ | GCGTTTTTTTTTTTTCGCCAGACGCGGTAAGACAAAATGTCGAACATCGTGAAGTGGCACCCCGTTGAGGTTTGAACCC |
| 4055SJ | GCGTTTTTTTTTTTTCGCCAGACGCGGTAAGACAAACGTTCGAACATCGTGAAGTGGCACCCCGTTGAGGTTTGAACCC |
| 4056SJ | GCGTTTTTTTTTTTTCGCACTTCCTGAAACGGGCACTGCACGTTTCAGCAAGTGTGGCACCCCGTTGAGGTTTGAACCC |
| 4057SJ | GCGTTTTTTTTTTTTCGCACTTCCTGAAACGGGCACTGGACGTTTCATCAAGTGTGGCACCCCGTTGAGGTTTGAACCC |
| 4058SJ | GCGTTTTTTTTTTTTCGCTTCTCACGCGCTAGCCTCCTGGATTCACGTGCTAAGTGGCACCCCGTTGAGGTTTGAACCC |
| 4059SJ | GCGTTTTTTTTTTTTCGCTTCTCACGCGCTAGCCTCGTGGATTCACGTGCTAAGTGGCACCCCGTTGAGGTTTGAACCC |
| 4060SJ | GCGTTTTTTTTTTTTCGCTTCACACGACGAAACGCCTCCCATTCAGGGCCAGGGTGGCACCCCGTTGAGGTTTGAACCC |
| 4061SJ | GCGTTTTTTTTTTTTCGCTTCACACGACGAAACGCCGGCCATTCAGGGCCAGGGTGGCACCCCGTTGAGGTTTGAACCC |
| 4066SJ | GCGTTTTTTTTTTTTCGCAAAGCACGCTACCGCCCCAGGCTCAACATCTAACTGTGGCACCCCGTTGAGGTTTGAACCC |
| 4068SJ | GCGTTTTTTTTTTTTCGCCTTCCAGCACACATCGCCTGAAAGACTAGTCACCAGTGGCACCCCGTTGAGGTTTGAACCC |
| 4069SJ | GCGTTTTTTTTTTTTCGCCTTCCAGCACACATCGGGTGAAAGACTAGTCACCAGTGGCACCCCGTTGAGGTTTGAACCC |
| 4071SJ | GCGTTTTTTTTTTTTCGCCTTCCAGCACACATCGATTAAAAGACTAGTCAGGAGTGGCACCCCGTTGAGGTTTGAACCC |
| 4072SJ | GCGTTTTTTTTTTTTCGCCTTCCAGCACACATCGATTAAAAGACTAGTTAATAGTGGCACCCCGTTGAGGTTTGAACCC |
| 4100SJ | GCGTTTTTTTTTTTTCGCCTTCGAGCACACATCGCCTGAAAGACTAGTTAATAGTGGCACCCCGTTGAGGTTTGAACCC |
